# Pericapsular nerve group (PENG) block combined with local infiltration analgesia is not superior to local infiltration analgesia for the management of postoperative pain after primary elective total hip arthroplasty: A prospective, randomized, controlled, single-blind trial

**DOI:** 10.1016/j.heliyon.2024.e33766

**Published:** 2024-06-27

**Authors:** Fabrice Ferré, Julien Rey, Laetitia Bosch, Rémi Menut, Anne Ferrier, Cyndie Ba, Caroline Halimi, Ioan Collinson, Bernard Tissot, François Labaste, Nicolas Reina, Vincent Minville

**Affiliations:** a Département d’Anesthésie-Réanimation, Hôpital Pierre-Paul Riquet, Centre Hospitalo-universitaire (CHU) Purpan, Toulouse, France; b Département d’Anesthésie-Réanimation, Centre Hospitalo-universitaire (CHU) Rangueil, Toulouse, France; c Département de chirurgie Orthopédique et Traumatologique, Hôpital Pierre-Paul Riquet, Centre Hospitalo-universitaire (CHU) Purpan, Toulouse, France

**Keywords:** Hip, Analgesia, Pericapsular nerve group block

## Abstract

**Background:**

Local infiltration analgesia (LIA) has been advocated for the pain management after total hip arthroplasty (THA). The analgesic benefits of an added pericapsular nerve group (PENG) block remain questionable.

**Methods:**

This randomized, single-blind trial enrolled patients undergoing elective THA under general anaesthesia and standardized postoperative analgesia. Patients were allocated to receive either a PENG block (20 mL of ropivacaine 0.475 %) combined with intraoperative LIA (PENG + LIA group, n = 32), or intraoperative LIA alone (LIA group, n = 32). The primary outcome was oral morphine equivalent (OME) consumption at day 1. Secondary outcomes were: pain scores at post anaesthesia care unit (PACU) discharge and on day 2, times for the Timed to Up and Go (TUG) test and measurement of adductor strength on day 1, and patients’ satisfaction using the EVAN-G questionnaire.

**Results:**

Compared with LIA alone, PENG + LIA resulted in similar OME consumption on day 1 (78 [51–91.5] mg vs 58 [30–80] mg respectively, median difference (95%CI) of −17 (−34 to 1) mg, p = 0.09). Pain scores and morphine consumption were not different between groups at any time point. TUG and thigh adduction tests were similar between LIA and PENG + LIA groups (respectively 35 [25–48.5] vs 31.5 [19.5–46.5] sec, p = 0.39; and 105 [85–150] vs 100 [80–125] mmHg, p = 0.61). No difference in the patients’ satisfaction was found.

**Conclusion:**

The addition of a PENG block to large-volume LIA did not significantly improve the analgesia for elective THA in the setting of an adequate basic postoperative analgesia regimen. The results of the lower limb functional tests confirmed the PENG block to be motor-sparing.

## Highlights

• **What is already known on this topic**.

The pericapsular nerve group (PENG) block is a novel ultrasound-guided block of the articular branches of the anterior hip capsule that provides i) a reduction in pain for patients with hip fractures, and ii) better pain relief after THA surgery compared to no block.

• **What study adds**

Adding a PENG block to a postoperative analgesia regimen including large-volume of local infiltration analgesia does not reduce the postoperative opioid consumption of patients scheduled for elective THA under general anaesthesia. On day 1, the lower limb motor function is not impaired by the PENG block.

• **How this study might affect research, practice or policy**

More data are needed to identify i) the intraoperative analgesic benefits of the PENG block placed before the surgery, and ii) the postoperative analgesic benefits of a ‘rescue’ PENG block done in the PACU for patients with intense immediate postoperative pain despite local infiltration analgesia.

## 1 Introduction

Total hip arthroplasty (THA) is one of the most common joint replacement surgeries with more than 100,000 eligible patients every year in France [1]. Implementation of a perioperative pain management protocol combined with early mobilization for THA patients could shorten the length of stay, accomplish therapy goals sooner and might also reduce the likelihood of complications after THA [[2], [3], [4]]. Pain management is therefore considered as a priority for the quality of patients’ functional recovery [5]. In this setting, opioids are the rescue analgesics most commonly used to treat moderate to severe postoperative pain in patients undergoing THA, but their side effects impair early rehabilitation [5].

A multimodal analgesia regimen including regional analgesia reduces morphine consumption (aka ‘opioid sparing effect’) and decreases postoperative pain scores. Thus, numerous regional analgesia techniques are described in the literature and are heterogeneously proposed to patients undergoing hip surgery. However, their analgesic benefits must be balanced by the resulting motor weakness (e.g., quadriceps paresis induced by the femoral nerve block) which may compromise patients' recovery [6] and increase the risk of falls [7]. Hence, peripheral nerve blocks are not routinely recommended to prevent postoperative pain after elective THA [8].

Another analgesic strategy would be to use large doses of local anaesthetics to infiltrate the surgical site. Indeed, compared with placebo, intraoperative periarticular injection of local anaesthetics, aka local infiltration analgesia (LIA), has shown better pain relief and a decrease in opioid consumption after THA with no risk of undesirable lower limb muscle weakness [8,9]. However, the evidence of the benefit of LIA in total hip arthroplasty is conflicting. It is not considered standard practice by all, nor is it routinely recommended [8].

A recent anatomical study on anterior hip capsule innervation led to the identification of relevant landmarks to target the hip articular branches of the femoral, obturator and accessory obturator nerves [10]. Using this information, Giron-Arango et al. developed a novel ultrasound-guided block of the articular branches of the anterior hip capsule, also known as the PENG (Pericapsular Nerve Group) block [10]. Five consecutive hip fractured patients underwent a PENG block, and all of them reported significant dynamic pain relief. It is worth noting that contrary to single nerve blocks (e.g., femoral nerve block), the PENG block targets several hip articular nerves branches involved in the innervation of the anterior hip joint capsule. In addition, given that the technique targets only the articular sensory branches, no significant effect of the motor strength is expected - contrary to blocks such as the fascia iliaca compartment block or the femoral nerve block [11,12].

Recently, Kukreja et al. have evaluated the postoperative analgesic efficacy of the PENG block for patients undergoing elective THA under spinal anaesthesia [13]. In their study, the PENG block reduced opioid requirements and improved the quality of recovery when compared with no block. However, neither study group received local anaesthetic infiltration, and therefore one might argue that this merely showed that some local anaesthetic (in the form of a PENG block) was better than nothing.

The main objective of our study was to evaluate the analgesic efficacy of the PENG block in patients scheduled for elective THA under general anaesthesia. We hypothesized that the PENG block combined with LIA was superior to LIA alone by reducing cumulative morphine consumption in the first 24 postoperative hours. Our secondary objectives were to evaluate the impact on functional motor strength as well as the patients’ satisfaction.

## 2 Methods

### 2.1 Design of the study

This interventional, single-blind, randomized controlled trial was conducted at the Purpan University Teaching Hospital (Toulouse, France) from December 2020 to July 2021. Written informed consent was obtained from all subjects. Approval for this study was given by the West VI Ethics Committee on October 8, 2020 (RC31/20/0227). This trial was registered on ClinicalTrials.gov (NCT04650100).

### 2.2 Inclusion and exclusion criteria

All patients ≥18 years old scheduled for elective THA under general anaesthesia were eligible.

The exclusion criteria were: previous hip replacement surgery on the same side (revision surgery); chronic pain requiring long-term use of opioids; surgery performed under spinal anaesthesia; patient refusal; major spontaneous or acquired disorders of hemostasis; infection at the puncture site; allergy to local anesthetics; and pregnancy.

### 2.3 Randomization

Using computer-generated randomization (Stata Statistical Software, Release 14, StataCorp LP, College Station, TX, USA), patients were successively randomized and assigned to either the LIA or the PENG block + LIA group. Allocation numbers were sealed in envelopes and opened at the time of inclusion on the day of surgery by the anaesthetist who performed the regional anaesthesia. Patients were not blinded to their group assignment (regional anaesthesia puncture or not). Patients were asked to not reveal the result of their allocation. The physician who evaluated the outcome criteria did not know the results of the randomization. The overall anaesthetic management (notably, general anaesthesia) was performed by the anaesthetist responsible for the patient in the operating room who was not involved in performing the PENG block or collecting data, and who was thus blinded to the group assignment of the randomization. Furthermore, the surgeon and nurses in charge of the patient were blinded to the group assignment. In all cases, skin disinfection with povidone-iodine was carried out in the inguinal region to make it less obvious that a patient had not received a PENG block.

### 2.4 General anaesthesia

For all patients, intravenous (i.v.) access was established. Standard vital sign monitors were placed and oxygen was delivered through a facemask. Surgery was performed under general anaesthesia induced with propofol (2–3 mg/kg) and sufentanil (0.2–0.3 μg/kg). Patients were orotracheally intubated after a single i.v. bolus of cisatracurium (0.1–0.2 mg/kg) or atracurium (0.5 mg/kg). Each patient received a bolus of i.v. ketamine (0.15–0.3 mg/kg). Maintenance of anaesthesia was performed with continuous i.v. propofol (2–6 mg/kg/h). Analgesic maintenance was performed with a bolus of sufentanil (2.5–7.5 μg) if necessary (encouraged when heart rate and/or systolic blood pressure increased by 20 % from baseline). For prophylaxis of postoperative nausea and vomiting, 8 mg i.v. dexamethasone were administered to each patient at the time of induction of anaesthesia, following loss of consciousness. Patients’ lungs were mechanically ventilated with a tidal volume of 6 ml/kg ideal bodyweight.

### 2.5 Surgery

All surgeries were performed by the same surgeon (NR) using a posterolateral and minimally invasive approach [14]. The incision was made over the gluteus maximus muscle. After sectioning the piriformis muscle, the joint capsule was approached in a latero-superior position with an L-shaped incision. The implant was inserted after sectioning the neck and preparing the acetabulum. Then, the hip capsule was reinserted as well as the obturator muscles.

Intra- and periarticular infiltration was performed at the end of the procedure by the surgeon.

The LIA procedure was standardized according to our current practice and consisted of an injection of 80 ml of ropivacaine 2 mg/mL (for a total dose of 160 mg [15]) distributed equally between the intra-articular and periarticular areas (under the posterior capsule), intermuscular areas (under the rectus femoris, between the gluteus minimus and tensor fasciae lata muscles and under the vastus lateralis muscle) and the subcutaneous area.

### 2.6 Postoperative analgesia

The postoperative analgesic management was standardized. At the end of surgery, patients received 1 g i.v. paracetamol and 100 mg i.v. ketoprofen in the absence of any contra-indications.

In the post anaesthesia care unit (PACU), pain was evaluated by an 11-point numerical rating scale (NRS) where 0 is no pain and 10 is the worst pain imaginable. Intravenous morphine was administered when the NRS was >3 [16], with an initial bolus dose of 0.05–0.1 mg/kg. On PACU discharge, all patients received 1 g paracetamol every 6 h for 7 days; 100 mg sustained-release ketoprofen every 12 h for 5 days; 10 or 20 mg (respectively for patients with a body weight < and ≥80 Kgs) sustained-release oxycodone every 12 h for 5 days; and 5 or 10 mg (respectively for patients < and ≥80 Kgs) immediate-release oxycodone as required, maximum every 4 h in case of pain, for 5 days.

### 2.7 Ultrasound-guided PENG block

A premedication with i.v. sufentanil (5 μg) prior to the PENG block was left to the discretion of the anaesthetist. Regional anaesthesia was performed prior to the general anaesthesia by an experienced anaesthetist trained to perform the PENG block.

The PENG block was performed as described by Giron-Arango et al. [10]. A 3–8 MHz microconvex ultrasound probe (C35xp, Sonosite S-Nerve II, Fujifilm®) was placed in the transverse plane above the anterior inferior iliac spine, and then aligned with the pubic ramus by a 45° anticlockwise (for the right hip) or clockwise (for the left hip) rotation. This ultrasound image should identify the iliopubic eminence, the iliopsoas muscle and its tendon, the femoral artery and the pectineus muscle. An 80 mm 22 G needle (PAJUNK SonoPlex® cannula) was inserted in the plane of the ultrasound, lateral to the probe and directed medially. An aspiration test was performed prior to any injection to verify the absence of a possible intravascular position of the needle. Slow, fractionated injections were performed when the needle tip was positioned and visualised under the iliopsoas muscle tendon. Twenty ml of ropivacaine 4.75 mg/ml (total dose of 95 mg) were injected between the tendon anteriorly and the iliopubic eminence posteriorly to allow a medial diffusion of the solution.

### 2.8 Endpoints

Patients were followed-up for 2 days after surgery.

• The primary endpoint was the total morphine consumption in the first 24 h after surgery (i.e., at day 1) compared between groups. Consumption was expressed as oral morphine equivalent (OME) and calculated as follow: 1 mg iv oxycodone = 1 mg iv morphine = 3 mg OME, and 1 mg oral oxycodone = 2 mg OME.
• Worst postoperative pain scores at rest (NRS, 0 to10, where 0 is no pain and 10 is the worst pain imaginable) were recorded by structured interview at the end of the PACU stay (“What is the highest rest pain score you have experienced during your stay in the recovery room?”), between PACU and day 1 (“What is the highest resting pain score you have experienced since leaving the recovery room?”), and between day 1 and day 2 (“What is the highest rest pain score you have experienced since our last assessment at the 24th postoperative hour?”). In the event of a discrepancy between the patients' responses and the resting pain scores provided by the nurses during their various assessments, the highest pain score was finally retained.
• Postoperative opioid consumption was recorded for the PACU, 24 h (day 1) and 48 h (day 2) after surgery by chart review. All opioids were converted to OME for comparison.
• An evaluation of the pain potentially induced by the PENG block was collected using a 1–5 Likert scale (1, much less than expected; 2, less than expected; 3, as expected; 4, more than expected; 5, much more than expected) in response to the statement "I felt pain when I had my regional anaesthesia".
• Patient satisfaction of the perioperative period was evaluated with the Evaluation du Vécu de l’Anesthésie Générale (EVAN-G) questionnaire on day 1. The EVAN-G is a validated patient-reported outcome measure that includes 26 items structured in six dimensions (attention; privacy; information; pain; discomfort; and waiting) [17]. The score for each dimension was linearly transformed to a scale from 0 to 100, where 100 is the best possible level of satisfaction and 0 is the worst. The global index score was calculated as the mean of the scores for each dimension.
• Motor functional impairment was assessed by an investigator blinded to the group allocation and tested on day 1 by:
  o The Timed Up and Go (TUG) test. The TUG is usually recommended as a routine screening test for falls, notably in older patients. This test allows the assessment of complex and global functions of mobility and strength [18,19]. Briefly, the patient is seated on a chair, has to stand up, walk 3 m in front of him, turn back to his chair, and sit down [20]; the TUG test score is given in seconds. The test is considered normal if the time is less than 14 s.
  o The only way to effectively evaluate the obturator nerve function is to assess the adductor strength. As previously described, the isometric strength of the adductor muscles was measured via a sphygmomanometer [21]. Briefly, the patient was asked to perform a thigh adduction on a pressure bag inflated to 40 mmHg and placed on the medial part of the contralateral knee; the pressure generated (in mmHg) was measured as the adductor force.
• The collection of common side effects associated with morphine (nausea, vomiting, pruritus, acute urine retention requiring urinary catheterisation, delirium or coma) was performed in both groups in the PACU, as well as on day 1 and day 2 after surgery.

### 2.9 Sample size prediction

Data from the literature suggest that patients consume about 50 ± 20 mg of OME in the first 24 h after THA under general anaesthesia and LIA [22]. Our working hypothesis was that the addition of a PENG block would reduce morphine consumption by 30 % compared to the LIA alone (difference of means between groups = 15 mg). To demonstrate a significant difference in analgesic consumption between the PENG + LIA and LIA groups with a two-sided alpha risk set at 5 % and a power set at 80 %, 29 patients per group had to be analyzed. In order to compensate for possible inclusion errors and patients lost to follow-up (generally estimated at 10 %), 32 patients were planned to be included in each group. If 29 or more patients per group completed the analysis of the primary endpoint at day 1, no additional inclusion would be made to compensate for secondary exclusions (per-protocol analysis).

### 2.10 Statistical analysis

The normality of the data was verified using the Shapiro-Wilk test. Quantitative variables were expressed as median [interquartile range, IQR] or mean (±SD) as appropriate. Categorical variables were expressed as numbers (%). The comparison of continuous variables between the PENG + LIA and LIA groups was performed using the Mann-Whitney *U* test. Categorical variables were compared using the Chi^2^ or Fisher's exact tests. The time course of pain and morphine consumption were studied using a repeated measures ANOVA. Two factors and their interaction were studied: the group effect (i.e., PENG + LIA vs. LIA) and the time effect. Statistical analysis was performed using MedCalc software (version 12.6.1, MedCalc Software bvba, Ostend, Belgium; 2013). A p-value <0.05 was considered statistically significant.

## 3 Results

**Fig. 1:**
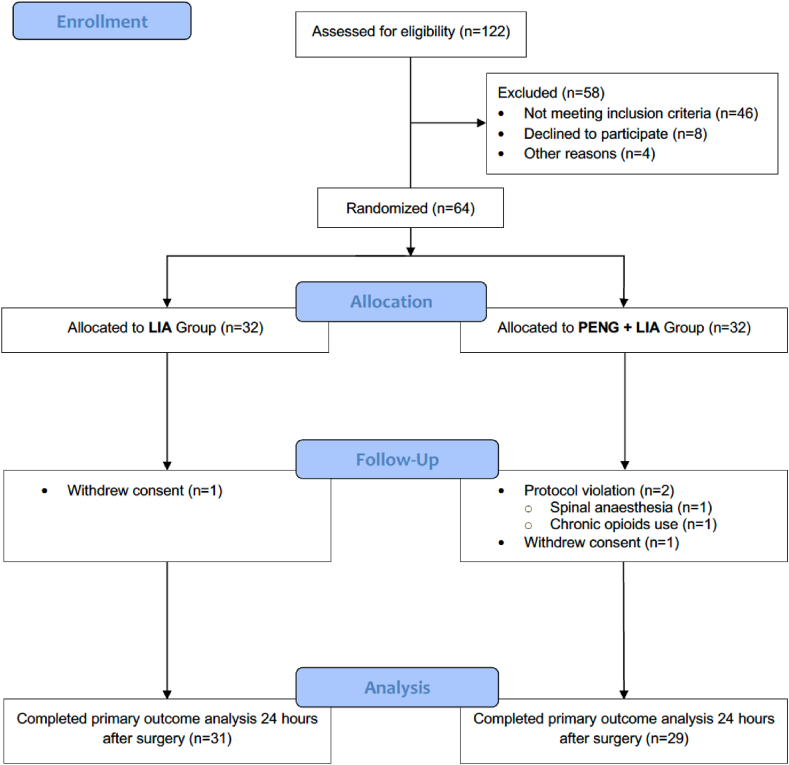
CONSORT flow diagram. Abbreviations: LIA, local infiltration analgesia; PENG, pericapsular nerve group.

Between December 2020 and July 2021, 64 patients were included in our study. The flow chart is shown in Fig. 1.

Demographic characteristics, including preoperative pain score, were comparable between groups (Table 1). Interestingly, the intraoperative sufentanil consumption was higher in the LIA group (25 [20–26.5] μg) than in the PENG + LIA group (20 [[15], [16], [17], [18], [19], [20], [21], [22], [23], [24], [25]] μg) with a median difference (95%CI) of −5 (−7.5 to 0) μg (p = 0.05 for comparison between groups) (Table 1).

**Table 1:** Demographic characteristics.

	LIA group (n = 31)	PENG + LIA group (n = 29)	p-value
Age (years)	60 [44.5–68.5]	61 [53–68]	0.59
Sex (female)	14 (45.2 %)	14 (48.3 %)	1
ASA score			0.65
• 1	16 (51.6 %)	13 (44.8 %)	
• 2	13 (41.9 %)	14 (48.2 %)	
• 3	2 (6.5 %)	2 (7 %)	
BMI (kg/m^2^)	24 [21.6–26.7]	26 [23.1–30.3]	0.05
Weight (Kg)	70 [63–77.5]	79 [67.5–85.5]	0.15
Past Medical History			
•Cardiovascular diseases	12 (38.7 %)	9 (31 %)	0.60
•Respiratory disease	2 (6.5 %)	2 (6.9 %)	1
•Chronic renal disease	3 (9.7 %)	3 (10.3 %)	1
•Diabetes	4 (12.9 %)	6 (20.7 %)	0.50
Surgery side			
•Right	17 (54,8 %)	15(51,7 %)	
•Left	14 (45,2 %)	14 (48,3 %)	
Indication for surgery			
•Osteoarthritis	28 (90.3 %)	27 (93.1 %)	
•Others	3 (9.7 %)	2 (6.9 %)	
Preoperative pain (NRS, 0–10)	2 [0–3]	1 [0–3.25]	0.92
Intraoperative ketamine (mg)	25 [15–38.75]	30 [[20], [21], [22], [23], [24], [25], [26], [27], [28], [29], [30]]	0.35
Intraoperative sufentanil (μg)	25 [20–26.5]	20 [[15], [16], [17], [18], [19], [20], [21], [22], [23], [24], [25]]	**0.05**

Data are expressed as median [IQR] or number (%).

LIA: local infiltration analgesia; PENG: pericapsular nerve group; ASA: American Society of Anesthesiology; BMI = body mass index; NRA: numerical rating scale.

### 3.1 Postoperative oral morphine equivalent (OME) consumption

OME consumptions are available in Table 2. OME consumptions at day 1 were not statistically different between groups: 78 [51–91.5] mg *vs*. 58 [30–80] mg for the LIA and PENG + LIA groups respectively (p = 0.09 for comparison between groups).

**Table 2:** Postoperative morphine consumption and pain scores.

	LIA group (n = 31)	PENG + LIA group (n = 29)	p-value
OME consumptions			
• In the PACU	18 [0–30]	12 [0–21]	0.14
•Between PACU and day 1	60 [30–80]	40 [20–60]	0.25
•At day 1	78 [51–91.5]	58 [30–80]	0.09
•Between day 1 and day 2	40 [20–60]	35 [10–60]	0.57
•At day 2	104 [75–139.5]	90.5 [61–124]	0.24
Worst postoperative pain scores at rest (NRS,/10)			
•In the PACU	4 [[3], [4], [5], [6]]	4 [[2], [3], [4], [5]]	0.34
•Between PACU and day 1	3 [[2], [3], [4], [5]]	3 [[2], [3], [4]]	0.97
•Between day 1 and day 2	4 [[2], [3], [4], [5]]	3 [0.5–3]	0.11

Data are expressed as median [IQR].

LIA: local infiltration analgesia; PENG: pericapsular nerve group; OME: oral morphine equivalent; PACU: post anaesthesia care unit; NRS: numerical rating scale.

The Hodges-Lehmann median difference (95%CI) in OME consumption at day 1 between PENG + LIA and LIA groups was −17 (−34 to 1) mg.

Similarly, OME consumption in the PACU (p = 0.14), between postoperative day 1 and day 2 (p = 0.57), and the total amount of OME consumption on postoperative day 2 (p = 0.24) were not statistically different between the groups (Table 2).

**Fig. 2:**
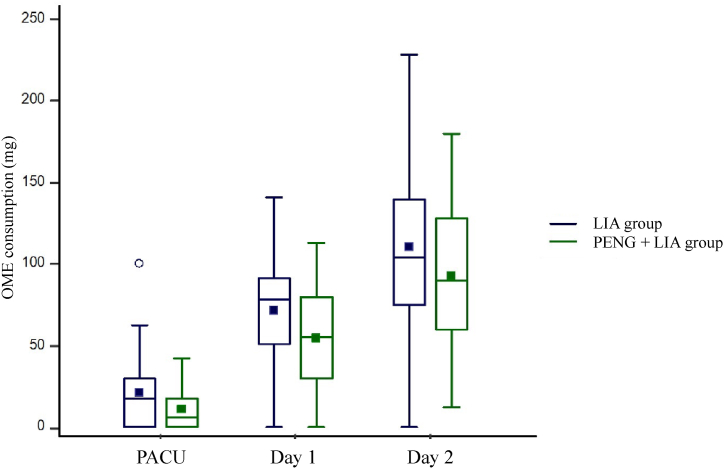
Evolution over time of oral morphine equivalent consumption in the LIA and PENG + LIA groups and their comparison between groups. No significant difference between groups was observed (p = 0.06). Abbreviations: OME, oral morphine equivalent; PACU, post anaesthesia care unit; LIA, local infiltration analgesia; PENG, pericapsular nerve group.

Fig. 2 illustrates the evolution over time of OME consumptions in the LIA and PENG + LIA groups. A significant difference between measurements (i.e., ‘time effect’) was observed (p < 0.001) with no significant difference between groups (p = 0.06). The difference between time measurements did not depend on group allocation (p = 0.62).

### 3.2 Postoperative pain

Maximum postoperative pain scores (i.e., maximum pain experienced since the last assessment) were not statistically different between groups, neither in the PACU (p = 0.34), nor between PACU and day 1 (p = 0.97), or between day 1 and day 2 (p = 0.11) (Table 2).

### 3.3 Secondary endpoints

The times for the TUG tests were not statistically different between LIA and PENG + LIA groups (35 [25–48.5] *vs.* 31.5 [19.5–46.5] sec respectively, p = 0.39 for the comparison between groups) (Table 3).

**Table 3:** Motor function tests, patients’ satisfaction and incidence of opioids-related side effects.

	LIA group (n = 31)	PENG + LIA group (n = 29)	p-value
Motor function	(n = 25)	(n = 26)	0.39 0.61
• TUG test (sec)	35 [25–48.5]	31.5 [19.5–46.5]	
• Thigh adduction test (mmHg)	105 [85–150]	100 [80–125]	
LOS (days)	2 [2–2.75]	2 [2–2]	0.14
Pain during PENG block (Likert scale/5)	–	2 [[1], [2], [3]]	–
Rescue femoral nerve block in PACU	4 (12.9 %)	0 (0 %)	0.11
EVAN-G (0–100)	64 [60–72]	65 [61–70]	0.85
Patients with ketamine titration in PACU	4 (12.9 %)	1 (3.4 %)	0.35
Patients with opioids adverse effects			
• PACU	3 (9.6 %)	2 (7.4 %)	0.67
• At day 1	4 (12.9 %)	3 (10.7 %)	0.76

Data are expressed as median [IQR] or number (%).

LIA: local infiltration analgesia; PENG: pericapsular nerve group; TUG: timed up and go; LOS: length of hospital stay; PACU: post anaesthesia care unit; EVAN-G: Evaluation du Vécu de l’Anesthésie Générale (patients' satisfaction score).

The results of the isometric strength test of the adductor muscles of the thigh were not statistically different between LIA and PENG + LIA groups (105 [85–150] *vs.* 100 [80–125] mmHg, p = 0.61 for the comparison between groups) (Table 3).

Pain experienced during the PENG block was less than expected (Table 3). Assessments and comparisons between groups of length of hospital stay, patient satisfaction of the perioperative period surrounding general anaesthesia, use of a rescue analgesic femoral nerve block in the PACU and morphine adverse effects are shown in Table 3. None of the comparisons were statistically significant (see Table 3 for p-values).

One patient experienced a serious adverse event (epileptic seizure) at day 1 for which the local anaesthetic was deemed not responsible (brain haemorrhage identified on CT scan).

## 4 Discussion

Determining the ideal peripheral nerve block for THA has proved challenging based on the complex innervation of the hip and the desire to avoid postoperative weakness. Through this randomized controlled trial carried out in primary elective total hip replacement surgery under general anaesthesia, we demonstrated that an added PENG block to large-volume surgical LIA did not reduce morphine consumption on postoperative day 1. In the setting of an adequate basic postoperative analgesia regimen, longitudinal evaluation of morphine consumptions and pain scores did not show statistically significant differences when comparing LIA and PENG + LIA groups.

### 4.1 Postoperative morphine consumptions and pain scores

Our results demonstrated that, in the context of a standardised postoperative analgesic regimen (including acetaminophen, NSAIDs and sustained-release opioids), a PENG block combined with LIA is not superior to LIA alone in the management of postoperative pain after elective total hip replacement surgery under general anaesthesia.

Our results are not in agreement with previously published data. In a retrospective study including 123 patients undergoing elective THA, Mysore et al. found a benefit from an added PENG block to LIA [23]. In their study, a reduction in OME consumption (mean difference −18 mg, 95 % CI: −29.3 to −6.7 mg), as well as a reduction in pain scores at postoperative day 1 were identified [23]. It's worth noting that the LIA performed by the surgeons was done using 20–40 mL of 0.25 % bupivacaine for all patients. Similarly, Zheng et al. have recently advocated the analgesic superiority of an added PENG block to LIA when compared with LIA alone for patients undergoing elective THA under general anaesthesia [24]. In their randomized placebo-controlled trial, the authors identified a maximum pain score reduction of 1.9 points (95%CI -3.3 to −0.5, p < 0.01) in PACU for patients allocated to the PENG + LIA group. Again, small volumes (20 ml of ropivacaine 0.5 %) were used for surgical LIA [24]. We believe that the difference identified by the authors in favour of the PENG + LIA could be explained by the small volume of local anaesthetics used for LIA. On the other hand, the negative result in our study might be related to the large volume of local anaesthetic used for LIA (in line with standard practice where volumes usually range from 60 to 170 mL [15,25]), which could have increased the analgesic benefits of the surgical LIA and attenuated the difference between groups. This assumption would encourage the infiltration of large volumes of local anaesthetics when LIA is used [8,26], while taking care not to exceed the permitted total doses of local anaesthetics (4 mg/kg of ropivacaine [27]) to prevent local anaesthetic systemic toxicity. We believe that 100 ml of ropivacaine 0.2 % (total dose 200 mg) would be an appropriate (effective and safe) choice for LIA in this setting [28].

### 4.2 Motor sparing effect of the PENG block

In our study, the PENG block did not alter the motor function required for standing and walking (TUG test) or for thigh adduction. To the best of our knowledge, our study is the first to evaluate the impact of the PENG block on the TUG test that allows the assessment of complex and global gait function. Our results are in agreement with those published by Pascarella et al. where the time to first walk was shorter in the PENG block than the control group [29]. It is worth noting that a significant correlation exists between gait function (e.g., gait speed and TUG test) and the quadriceps strength, as demonstrated for patients undergoing total knee arthroplasty [30]. For patients undergoing elective THA, Zheng et al. showed that there were no significant differences in quadriceps strength levels between PENG + LIA and LIA groups, on either the operated side or the non-operated side [24]. For hip fracture patients, the PENG block has been shown to be superior compared to the femoral nerve block for preserving the quadriceps function assessed in the PACU and at day 1 [12]. Another recently published randomised controlled trial confirmed the motor sparing effect of the PENG block when compared to a supra-inguinal fascia iliaca block [11]. Another recent study comparing PENG and LIA, with quadriceps weakness being the primary outcome, found no difference in motor block between groups [31].

On the other hand, inadvertent quadriceps weakness following the PENG block has been described. Three main hypotheses have been evoked to explain such an effect: an injection medial to the iliopubic eminence (resulting in diffusion to the femoral nerve), the use of too large volumes, or an intramuscular injection into the iliopsoas muscle [11,32].

Although no analgesic benefit was demonstrated in our study, our functional results suggest that a PENG block could be an interesting alternative to a femoral nerve block when regional analgesia becomes necessary to manage intractable postoperative pain in the PACU. This could, for example, be used for patients where the surgical LIA may be ineffective (e.g., because inadequately performed). Finally, the study of a PENG block for patients with chronic pain and/or opioid-dependency may be of significant interest [33].

### 4.3 Limits

First, one of the main pitfalls was the systematic delivery of sustained-release opioids as part of our standardized postoperative analgesic regimen. A certain number of patients potentially did not need postoperative administration of opioids. Indeed, 2 patients in the LIA group and 3 patients in the PENG + LIA group did not want to take the prescribed opioids as they considered that they had sufficient pain control. The exact number of patients who may have taken opioids without a clear need and their distribution between groups remain to be determined. Opioids should be reserved as rescue analgesics in the postoperative period [8].

Second, the number of patients requiring a rescue analgesic femoral nerve block in the PACU was higher in the LIA than in the PENG + LIA group (without reaching statistical significance). Theoretically, this strategy may have reduced the patients’ subsequent morphine consumption, contributing to the negative results of this study. However, additional analyses without patients having postoperative femoral nerve blocks did not change the result of the primary endpoint. Third, our results may not be applicable for THA done via the anterior surgical approach. Indeed, the PENG block is supposed to involve the nerves of the anterior side of the hip joint whereas the posterior side is innervated by the branches divided from the sciatic and superior gluteal nerves and the nerve to the quadratus femoris. Thus, a PENG block could be more effective in the pain management after THA using the anterior approach, in which the posterior component of hip joint capsule is not manipulated.

Fourth, the assessment of the intraoperative analgesic benefit (i.e., intraoperative morphine sparing effect and nociception) of the PENG block realized before the surgery, as suggested by the (albeit small) difference in intraoperative sufentanil consumption between groups, was not specifically studied.

Finally, the near-significance in some results suggests that the study might have been underpowered. A larger sample might reveal statistically significant differences. It is crucial for readers to consider these limitations when interpreting the study's findings and their applicability to broader clinical practice.

## 5 Conclusion

To conclude, a preoperative PENG block had no additional postoperative analgesic benefits for elective THA in the setting of an adequate basic postoperative analgesia regimen including large-volume LIA. Functional test results suggest that PENG block spares motor function. Additional investigations are required to elucidate the postoperative analgesic benefits of a ‘rescue’ PENG block done in the PACU for patients with postoperative pain despite LIA.

## Funding statement

Support was provided solely from department sources. This work should be attributed to the Département d’Anesthésie Réanimation et Médecine Périopératoire, Centre Hospitalo-Universitaire (CHU) Purpan, Toulouse, France.

## Data availability statement

Data associated with our study has not been deposited into a publicly available repository. Anonymized data will be available on request from the corresponding author.

## CRediT authorship contribution statement

**Fabrice Ferré:** Writing – review & editing, Writing – original draft, Supervision, Methodology, Investigation, Formal analysis, Data curation, Conceptualization. **Julien Rey:** Methodology, Funding acquisition, Formal analysis, Conceptualization. **Laetitia Bosch:** Supervision, Project administration, Methodology, Data curation, Conceptualization. **Rémi Menut:** Supervision, Project administration, Methodology, Investigation, Data curation, Conceptualization. **Anne Ferrier:** Project administration, Formal analysis, Data curation, Conceptualization. **Cyndie Ba:** Validation, Methodology, Funding acquisition, Data curation, Conceptualization. **Caroline Halimi:** Visualization, Funding acquisition, Formal analysis, Data curation, Conceptualization. **Ioan Collinson:** Methodology, Investigation, Formal analysis, Data curation, Conceptualization. **Bernard Tissot:** Supervision, Methodology, Funding acquisition, Formal analysis, Data curation, Conceptualization. **François Labaste:** Writing – original draft, Validation, Methodology, Conceptualization. **Nicolas Reina:** Writing – original draft, Validation, Formal analysis, Data curation, Conceptualization. **Vincent Minville:** Writing – review & editing, Writing – original draft, Supervision, Methodology, Formal analysis, Conceptualization.

## Declaration of competing interest

The authors declare that they have no known competing financial interests or personal relationships that could have appeared to influence the work reported in this paper.
